# Longitudinal Dietary Intake of Young People With a First Episode of Psychosis From the Time of Presentation to Six Months Follow‐Up

**DOI:** 10.1111/eip.70226

**Published:** 2026-07-28

**Authors:** Brian O'Mahony, Andrew Thompson, Adele Morrin, Brian O'Donoghue

**Affiliations:** ^1^ School of Medicine University College Dublin Dublin Ireland; ^2^ Centre for Youth Mental Health University of Melbourne Melbourne Australia; ^3^ Orygen Youth Health Parkville Victoria Australia; ^4^ Milltown Mental Health Services Dublin Ireland; ^5^ Department of Psychiatry St Vincent's University Hospital Dublin Ireland

**Keywords:** antipsychotic agents, diet, feeding behaviour, longitudinal studies, psychotic disorders, weight gain

## Abstract

**Background:**

Antipsychotic medications stimulate appetite and weight gain, contributing to high obesity rates in people with psychotic disorders. It is unknown if these dietary effects habituate over time. This study aimed to determine the dietary intake of young people with a first‐episode psychosis at the time of presentation and at three‐ and six‐months of follow‐up.

**Methods:**

We assessed dietary patterns at baseline, 3 months, and 6 months in participants diagnosed with first‐episode psychosis who had minimal prior antipsychotic exposure. We analysed data from the PHAstER randomised‐controlled trial, assessing changes in food consumption and Australian Dietary Guideline compliance using signed‐rank tests with corrections for multiple testing.

**Results:**

Of the 77 PHAstER study participants, 70 participants (91%) provided dietary data at baseline and 46 (60%) at 3 and 6 months. Dietary intake patterns and compliance with dietary guidelines remained consistent across all three timepoints. At baseline, few met recommended intake of three major food groups: vegetables (13%), dairy (20%) and fruit (24%). 64% of participants had a high intake of discretionary food (food high in saturated fat/sugars). At baseline, but not at three‐ or six‐months, higher education levels were associated with healthier dietary patterns.

**Conclusion:**

Participants consistently reported unhealthy diets that did not adhere to guidelines, with no significant change over 6 months. This study reinforces the inequity of dietary health in people with psychosis, particularly those with lower levels of education. Dietary advice and support represent interventions with high marginal gain in this cohort, and should be part of routine clinical care.

## 1 Introduction

Multiple international studies have reported that people with psychotic disorders have an elevated risk of obesity (Afzal et al. 2021). This increased weight drives dysfunction in metabolic health, such as lipid and glucose regulation (Pillinger et al. 2019; Dali‐Youcef et al. 2013; Fernández‐Verdejo and Galgani 2022), which is in turn a primary driver in the 15–20 year reduction in life expectancy people with psychotic disorders experience (Laursen 2011; Firth et al. 2019). Factors known to predispose individuals to overweight and obesity, including genetic factors, sedentary behaviour, social isolation, and negative discrimination, are common in people with psychosis (Stubbs et al. 2016; Manu et al. 2015).

Although people with psychotic disorders appear predisposed to metabolic dysfunction prior to treatment (Perry et al. 2016; Pillinger et al. 2019), this metabolic dysfunction is accentuated by treatment with antipsychotic medication, as is the associated weight gain (Pillinger et al. 2020). Antipsychotics exert deleterious effects on metabolic health through their widespread effects on neuromodulators (Carli et al. 2021). A major factor in their propensity to cause weight gain is the increased appetite they induce (Roerig et al. 2005; Ballon et al. 2018), through antagonism of histamine‐1 and serotonin 2_c_ receptors (Carli et al. 2021).

Previous studies assessing dietary patterns in people with psychosis have reported that these individuals typically have an unhealthy diet, with lower consumption of vegetables and higher consumption of saturated fat, processed foods, sodium, and overall calories than the general population (Hahn et al. 2014; Nenke et al. 2015; Teasdale et al. 2019). An important question is whether this effect on dietary intake changes over time. Medication side effects can sometimes display habituation (becoming less prevalent and bothersome over time), as in the sedative effects of antipsychotics and some side effects of antidepressants (Nomura et al. 2025; Braund et al. 2021). A similar diminishing effect on appetite would mean that the initial surge in appetite and carbohydrate craving would lessen over time, which could offer some explanation for the peak of the rate of weight gain seen in the first 3 months of people with a First Episode Psychosis (FEP) taking antipsychotics (Alvarez‐Jimenez et al. 2008; Pérez‐Iglesias et al. 2014).

Most studies linking psychosis and unhealthy diet are of cohorts with chronic/enduring psychosis, well beyond the point where this possible habituation could be examined. Therefore, FEP cohorts, who have not taken antipsychotics previously, are the cohort in which this question of dietary change over time can be appropriately examined. Previous studies which have reported unhealthy diet in FEP collected data cross‐sectionally and retrospectively, but not longitudinally (Saugo et al. 2020; Teasdale et al. 2018).

We aimed to answer this question by using data from a study that longitudinally tracked food consumption in individuals who had newly started antipsychotic medication and aimed to investigate if there was any change in their dietary pattern as their treatment continued.

## 2 Methods

### 2.1 Study Design

This study examines a secondary outcome of the Physical Health Assistance in Early Psychosis (PHAstER) study. The PHAstER study was a randomised‐controlled trial which aimed to determine whether the integration of a physical health nurse in the care of young people (aged 15–24 years) with first‐episode psychosis could prevent clinically significant weight gain (O'Donoghue et al. 2022). No difference was found in the physical health outcomes of the people allocated to the intervention group (the addition of a physical health nurse) and those allocated to treatment as usual, and therefore the combined group was examined in this secondary analysis study.

### 2.2 Setting

The PHAstER study took place at EPPIC, an early intervention for psychosis service that is part of Orygen, a youth mental health service for young people aged 15–24 years residing in North‐West Melbourne, Australia. The service covers a total catchment area of over 1 million residents, of whom approximately 200 000 were aged between 15 and 24 years.

### 2.3 Participants

The PHAstER study included participants aged 15–24 years, diagnosed with FEP, and who had fewer than 30 days of cumulative exposure of the minimum effective dose of an antipsychotic medication. All participants received care from a case manager and psychiatrist within the same FEP service, while those in the intervention group received additional support from a physical health nurse.

### 2.4 Instruments

Participants in this study provided data on their dietary intake using a *modified version of a screening tool for nutritional risk* (Appendix A). Participants provided data on the frequency of their intake of a variety of food categories. Participants completed this food questionnaire with the aid of a research assistant.

We benchmarked participant intake against the Australian Dietary Guidelines (National Health Medical Research Council 2013). As our questionnaire differed in format from these guidelines, we transformed our data to match the guidelines as shown in Table 1. We excluded the ‘lean meat and alternatives’ (protein) food group from this compliance analysis, as the available dietary questionnaire items were insufficient to capture total daily intake from all sources (e.g., poultry was not included).

**TABLE 1:** Australian dietary guidelines and their definitions from our questionnaire.

Food group	Australian guideline recommendation	Study operationalisation (compliance criteria)
Dairy	2.5–4 servings	**Compliance:** ≥ 2.5 servings per day (based on low‐fat dairy item).
Fruit	2 servings per day	**Proxy:** Selected highest frequency category (‘Every day or almost every day’).
Grains	Adequate wholegrain intake (6–7 servings per day)	**Proxy:** Reported consuming wholegrain bread OR wholegrain cereals at the highest frequency (‘More than twice per week’).
Vegetables	5–6 servings per day	**Calculated total:** Sum of quantitative servings at the main meal + estimated average daily servings of legumes.
		*Note: An alternative method adding frequency‐based salad/root vegetables was tested but yielded identical compliance results*.
Discretionary foods	Limit intake (no specific quantity)	**Strict method:** Selected ‘Never’ or ‘Less than once per week’ for all discretionary items.
		**Lenient method:** Did not select ‘More than twice per week’ for any discretionary items.^a^

^a^ Discretionary items defined as: soft drinks, chocolate, crisps, chips, cakes, deli meats, and takeaway.

### 2.5 Data Analysis

For our primary analysis, we used only the complete dataset of dietary questions. For a sensitivity analysis we handled missing dietary data using multiple imputation by chained equations (MICE) in R version 4.5. We imputed missing 3‐month (*n* = 475) and 6‐month (*n* = 562) dietary responses using predictive mean matching across 10 datasets with 10 iterations. We configured the predictor matrix to ensure each follow‐up dietary question was predicted by its baseline value, temporal siblings, sex, and age.

We set the significance level (α) at 0.05 and adjusted for multiple testing using the Benjamini‐Hochberg (FDR) correction. We assessed changes in dietary patterns at baseline, 3 months, and 6 months by using the Wilcoxon signed‐rank V statistic. We performed a sensitivity analysis by assessing whether the above tests differed after controlling for the RCT group assignment.

### 2.6 Ethical Approval

The study received ethical approval from the Melbourne Health Human Research Ethics Committee (approval number HREC/18/MH/77).

## 3 Results

### 3.1 Description of Participants

The main RCT included 77 patients, 70 of whom completed the dietary questionnaire at baseline. Table 2 displays the demographics of study participants. The mean age of participants was 19.4 years, and the majority were unemployed (68%) and living with their family of origin (82%). At 3 months, 50 young people provided dietary data (47 participants with full completion and three with partial completion) and 46 provided dietary data at 6 months (44 with full completion and 2 with partial completion). People who did not complete the questionnaire at baseline differed in employment status, with a higher proportion of students not completing the questionnaire (*p* = 0.006). There were no differences in baseline characteristics between those who completed questionnaires and those who did not complete questionnaires at 3 months or at 6 months.

**TABLE 2:** Patient demographics at baseline.

Characteristic	Overall	Completed	Not/Partially completed	p^b^
	*N* = 77^a^	*N* = 70^a^	*N* = 7^a^	
Age (years)	19.442 (3.420)	19.629 (3.461)	17.571 (2.440)	0.050
Sex				0.4
Male	39 (51%)	37 (53%)	2 (29%)	
Female	36 (47%)	31 (44%)	5 (71%)	
Intersex	2 (2.6%)	2 (2.9%)	0 (0%)	
Migrant	15 (19%)	15 (21%)	0 (0%)	0.3
Partner				0.3
Single	60 (78%)	53 (76%)	7 (100%)	
In a relationship	17 (22%)	17 (24%)	0 (0%)	
Accommodation				> 0.9
Living with family of origin	63 (82%)	56 (80%)	7 (100%)	
Rented room	3 (3.9%)	3 (4.3%)	0 (0%)	
Rented flat/house	4 (5.2%)	4 (5.7%)	0 (0%)	
Owned flat/house	1 (1.3%)	1 (1.4%)	0 (0%)	
Boarding house/hotel	1 (1.3%)	1 (1.4%)	0 (0%)	
Homeless	4 (5.2%)	4 (5.7%)	0 (0%)	
Other	1 (1.3%)	1 (1.4%)	0 (0%)	
Education level				0.9
Primary education	0 (0%)	0 (0%)	0 (0%)	
Secondary education (Year 7–10)	12 (24%)	10 (23%)	2 (29%)	
Secondary education (Year 11–12)	18 (36%)	14 (33%)	4 (57%)	
TAFE	6 (12%)	6 (14%)	0 (0%)	
University undergraduate	10 (20%)	9 (21%)	1 (14%)	
University postgraduate	2 (4.0%)	2 (4.7%)	0 (0%)	
Other	2 (4.0%)	2 (4.7%)	0 (0%)	
Unknown	27	27	0	
Employment status				0.006
Unemployed	40 (68%)	39 (72%)	1 (20%)	
Employed part‐time	6 (10%)	6 (11%)	0 (0%)	
Employed full‐time	4 (6.8%)	4 (7.4%)	0 (0%)	
Student	9 (15%)	5 (9.3%)	4 (80%)	
Unknown	18	16	2	
Antipsychotic at baseline				0.4
No medication prescribed	6 (7.8%)	5 (7.1%)	1 (14%)	
Partial dopamine agonist only	31 (40%)	27 (39%)	4 (57%)	
Second generation only	40 (52%)	38 (54%)	2 (29%)	
Days of antipsychotic exposure	16.299 (9.713)	16.129 (9.685)	18.000 (10.614)	0.5
BPRS total score	58.667 (12.596)	58.569 (12.400)	59.571 (15.372)	0.8
Unknown	5	5	0	

Abbreviations: BPRS = Brief Psychiatric Rating Scale; TAFE = Technical and Further Education.

^a^ Mean (SD); *n* (%).

^b^ Wilcoxan rank sum test; Fisher's exact test.

### 3.2 Dietary Intake at Presentation (Within 4 Weeks of Initiation of Antipsychotic Medication)

At baseline, 64% of young people with a first episode of psychosis were consuming more discretionary foods (food not necessary for a healthy diet, and high in saturated fat/sugars) than recommended. 76% were not consuming sufficient fruit and 87% were not consuming enough vegetables. Less than half (44%) were consuming a sufficient amount of grains and only 20% were compliant with dairy intake recommendations.

Among discretionary foods, half of young people with a FEP were consuming more than recommended quantities of sugary drinks (28.5% more than two soft drinks per week and 21.5% once or twice a week) and 35.7% reported eating chocolate more than twice per week.

**FIGURE 1:**
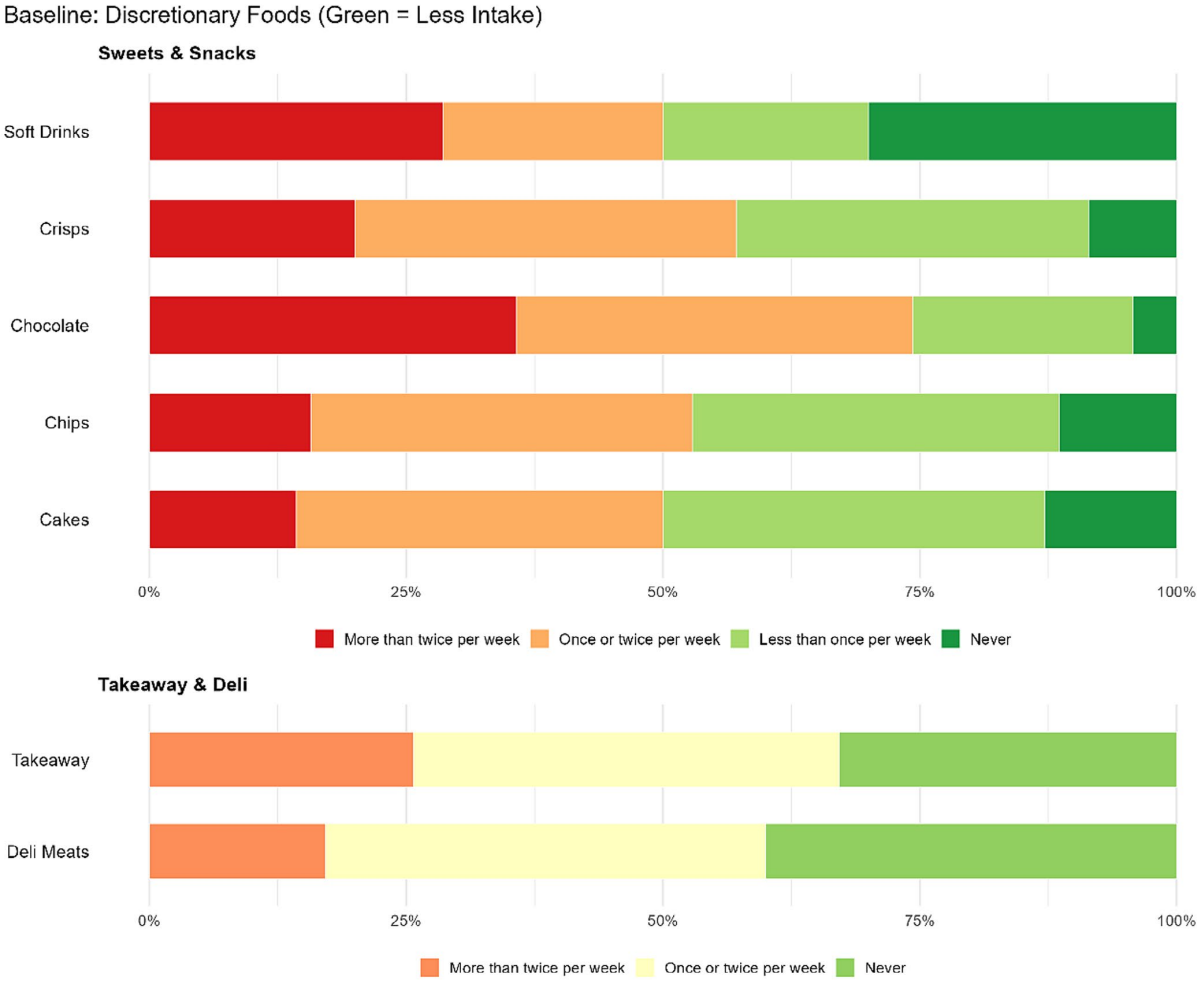
Consumption of discretionary foods at baseline.

**FIGURE 2:**
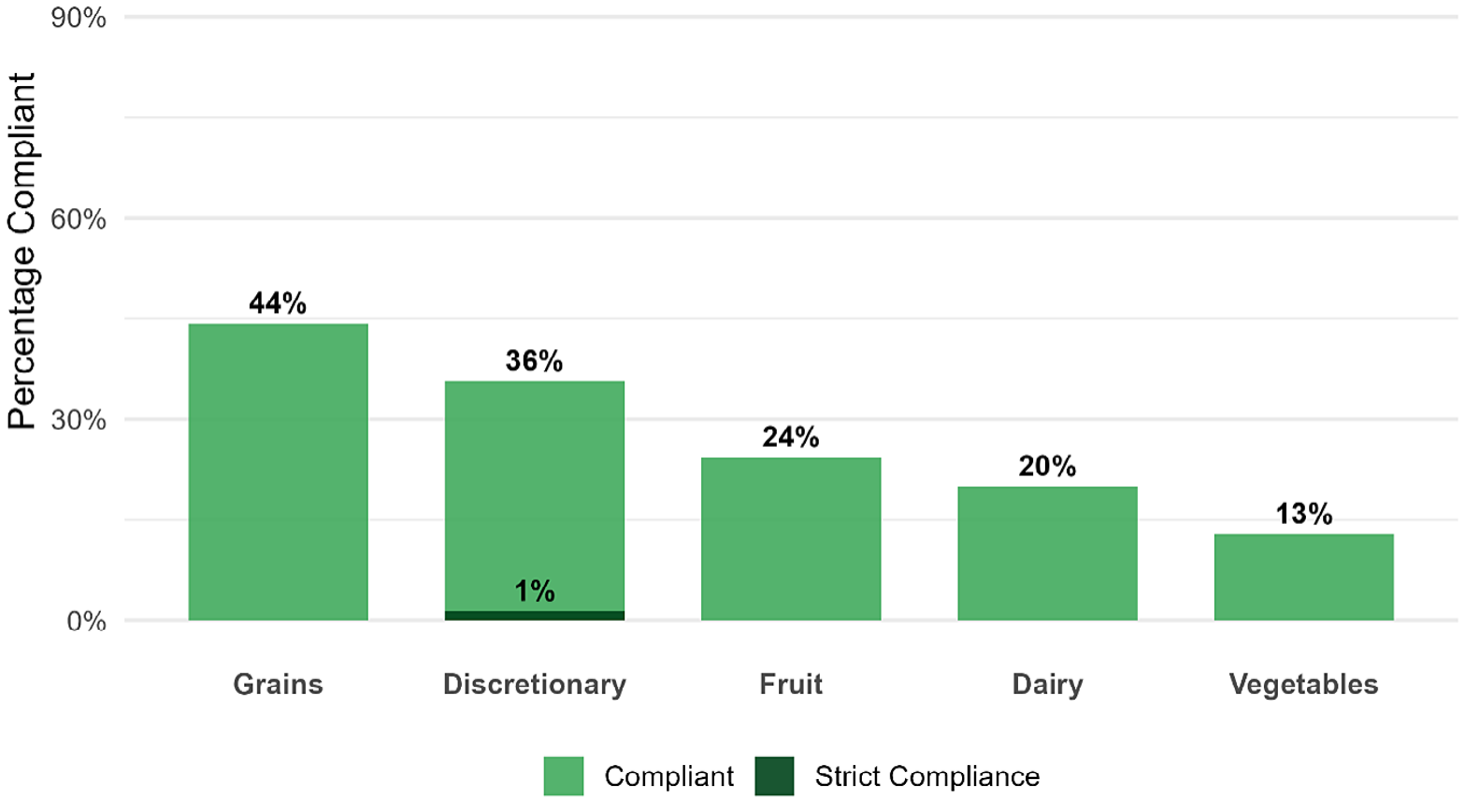
Percentage of people compliant with Australian Dietary Guidelines at baseline.

Dietary quality at baseline was generally low, as evidenced by the majority of patients not having the recommended intake of any of the food categories set out in the guidelines. Figure 1 displays participants' intake of discretionary food items at baseline, while Figure 2 shows estimated levels of compliance with the Australian Dietary Guidelines at baseline.

### 3.3 Comparison of Dietary Intake at 3‐and 6‐Month Timepoints

Patterns at the follow‐up timepoints were similar to those at baseline, although it should be noted that the 46 participants who reported 3‐month and 6‐month dietary intake represent a subset of the 70 who reported intake at baseline. At both 3 months and 6 months, the majority of participants again did not meet recommended consumption of any of the food categories set out in the guidelines. At 3 months, 63% were consuming more discretionary foods than recommended. 76% were not consuming sufficient fruit, and 96% not enough vegetables. Again, less than half (44%) were consuming a sufficient amount of grains, and only 16% were compliant with dairy intake recommendations. Dietary patterns at 6 months were broadly the same: 52% were eating more discretionary foods than recommended, and the majority were non‐compliant with recommendations for consumption of all groups; only 33% consumed adequate wholegrains, 17% consumed adequate dairy products, and 11% consumed enough fruit and vegetables.

Among discretionary foods, similar to baseline, at 3 months half of young people with a FEP were consuming more than recommended quantities of sugary drinks (28% more than two soft drinks per week and 22% once or twice a week) and 32% reported eating chocolate or sweets more than twice per week. At 6 months, 15.2% of participants reported consuming soft drinks more than twice per week, while 17.4% reported consuming soft drinks once or twice per week. At six months, 30.4% of young people reported consuming chocolate or sweets more than twice per week.

**FIGURE 3:**
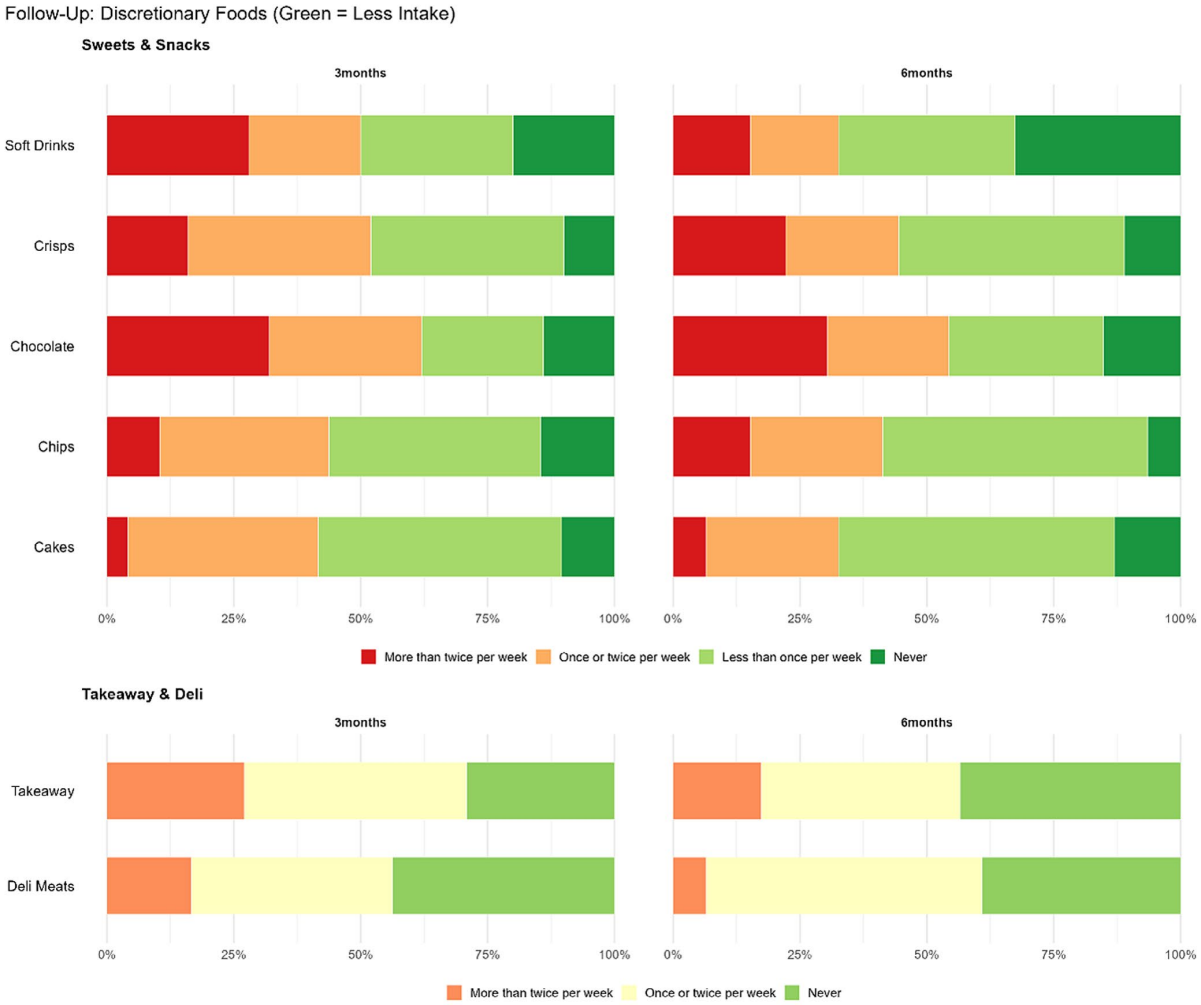
Consumption of discretionary foods at 3 months and 6 months.

**FIGURE 4:**
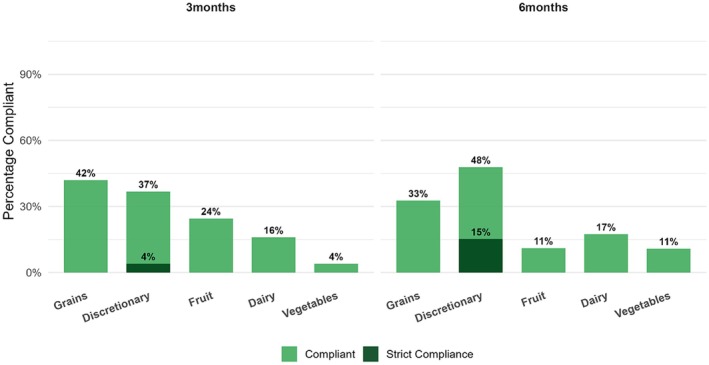
Percentage of people compliant with Australian Dietary Guidelines at follow‐up timepoints.

Figure 3 displays participants' intake of discretionary food items at 3 months and 6 months, while Figure 4 shows the estimated levels of compliance with the Australian Dietary Guidelines at these timepoints.

Intake of food categories and compliance with dietary guidelines were consistent across all three timepoints, with no difference in intake between baseline and 3 months or between baseline and 6 months reaching statistical significance, even prior to correcting for multiple testing. Appendix B displays figures for non‐discretionary food items at all three timepoints. Additionally, there was no difference in dietary patterns at the follow‐up timepoints between the group who received the PHAstER study intervention and those in the control group.

### 3.4 Clinical and Demographic Factors Associated With Dietary Intake

Intake of seven food groups differed by educational level at baseline, but not at 3 months or 6 months after applying the Benjamini‐Hochberg correction for multiple comparisons. The most significant difference was in the consumption of seafood (not fried) (*χ* ^2^ (5) = 25.468, *p*_*adj*_ = 0.002). There were also statistically significant differences in consumption of legumes (*χ* ^2^ (5) = 19.794, *p*_*adj*_ = 0.014), broccoli/cauliflower (χ^2^ (5) = 18.119, *p*_*adj*_ = 0.019), crisps (*χ* ^2^ (5) = 15.026, *p*_*adj*_ = 0.047), chips (χ^2^ (5) = 14.376, *p*_*adj*_ = 0.047), wholegrain cereals (*χ* ^2^ (5) = 14.249, *p*_*adj*_ = 0.047), and nuts (χ^2^ (5) = 13.790, *p*_*adj*_ = 0.049). Across these outcomes, people with higher education reported healthier dietary patterns. No other dietary items showed statistically significant differences across education levels (all *p*_*adj*_ > 0.05).

After adjusting for age, educational level remained a significant predictor for several dietary components at baseline. Specifically, nine food groups showed statistically significant differences (*p*_*adj*_ < 0.05) in models controlling for age, compared to seven in the unadjusted analysis. The strongest association remained the intake of non‐fried seafood (*F*(5) = 8.365, *p*_*adj*_ < 0.001), followed by legumes (*F*(5) = 5.741, *p*_*adj*_ = 0.001), broccoli/cauliflower (*F*(5) = 3.767, *p*_*adj*_ = 0.024), and salad (*F*(5) = 3.227, *p*_*adj*_ = 0.040). Significant differences also persisted for crisps, chips, wholegrain cereals, chocolate & sweets, and cakes (all *p*_*adj*_ < 0.05), while the previously significant association with nuts was attenuated (*p*_*adj*_ = 0.051). Across these adjusted models, higher educational attainment continued to be associated with healthier dietary patterns, indicating that the effect of education on diet is independent of participant age.

### 3.5 Sensitivity Analysis Using Multiple Imputation Methods

Our sensitivity analysis using multiple imputation methods supported the findings of our primary analysis. We observed no significant differences in intake between baseline and the three‐ or six‐month follow‐ups after adjusting for multiple testing. Pooled estimates for food consumption and compliance with Australian Dietary Guidelines, alongside statistical comparisons between timepoints, are presented in Appendix C (Table C1).

## 4 Discussion

### 4.1 Summary of Findings

To our knowledge, this is the first study to longitudinally assess the dietary patterns of people with FEP early in their treatment. Participants consistently reported more frequent intake of soft drinks and chocolate/sweets than of core food items such as vegetables, salad, seafood, or wholegrain cereals at all time points. We found no differences in dietary patterns at baseline (shortly after treatment initiation) compared to 3 months or 6 months into treatment. We thus failed to reject the null hypothesis that there is no difference in dietary intake as FEP progresses from its early stages to later in the course of the illness.

### 4.2 Comparison to Previous Literature

Our findings are in in keeping with previous reports of unhealthy dietary patterns of people with FEP. As per Australian Dietary Guidelines, the majority of participants reported inadequate consumption of wholegrains, and over three quarters reported inadequate consumption of fruits and vegetables, which is in keeping with previous Australian research (Hahn et al. 2014). Consumption among discretionary foods is common in Australian young people, but this is counterbalanced by a much higher level of healthier‐food consumption than the participants in our study (Australian Bureau of Statistics 2023). Separate Lancet Commission reports have set out the need to limit intake of Ultra‐Processed foods (Monteiro et al. 2025) and address the gap in physical health between the general population and people with severe mental illness (Firth et al. 2019). Clearly, addressing the unhealthy diet of people with psychotic disorders represents an overlap of these two recommendations. This would be especially important to implement in FEP, the first point of contact for many with services. However, implementing such changes is challenging given the motivational and demographic challenges experienced by people with FEP (Arnautovska et al. 2022; James et al. 2025). As such, while advice on healthy eating (e.g., advising reduction of discretionary food items) presents an opportunity for services to intervene, nutrition interventions delivered by dietitians appear to carry greater benefit than those carried out by non‐dietitians (Rocks et al. 2022). Qualitative research has demonstrated how a lack of baseline cooking skills and knowledge of healthy diet can be transformed by dietitian support (Bogomolova et al. 2018). Dietitian input has been shown to reduce weight gain in people with FEP, albeit in studies with small samples, (Curtis et al. 2016; Teasdale et al. 2015) and should be aimed to be part of standard care for FEP. Widespread adoption of dietitian support for people with FEP, as recommended by the recent Lancet Commission report, may go some way in reducing the elevated risk of nutritional deficiencies and food insecurity seen in this cohort (Firth et al. 2018; Smith et al. 2024; Teasdale et al. 2025).

Our data also aligned with literature showing that people with lower levels of education consistently have a more unhealthy diet (European Food Information 2025). Of note, these differences were present at baseline but not at 3‐months and 6‐months. One explanation could be that the provision of a multidisciplinary team input, including dietitian support, reduced this inequity. Alternatively, drop‐outs could have meant that these timepoints lost power to detect differences. Nonetheless, given the existing inequity in dietary patterns and food security between people with differing levels of education, providing dietary advice to this population could yield the greatest benefit.

### 4.3 Possible Explanations for Findings

The lack of change in dietary patterns over the initial few months of treatment may indicate that antipsychotic medications exert a chronic effect on appetite and cravings. Our ‘baseline’ results are likely to reveal a diet influenced by the effects of antipsychotics, rather than the participants' pre‐existing baseline (before they began to experience psychosis or before the initiation of antipsychotics). Although the participants in this study were early in their treatment (less than 30 days of antipsychotic exposure), they had all commenced antipsychotics by the time of their baseline assessment. Thus, given qualitative research indicating that the effects of antipsychotics on appetite occur rapidly (Alkholy et al. 2025), these dietary changes may have already taken place.

### 4.4 Future Research

Sufficient evidence has now accumulated of an unhealthy diet in people with psychotic disorders, although the diets of this cohort prior to treatment with antipsychotics is an obvious avenue for future research. One possibility would be to retrospectively collect this data following onset of psychosis, but this would be prone to recall error. Another avenue would be to monitor the dietary patterns of ultra high‐risk of psychosis cohort before and after their initiation of antipsychotics. Of this group, 15% and 25% will transition to psychosis after 1 and 3 years, respectively (De Pablo et al. 2021). Collecting regular, but not necessarily frequent, dietary data prior to transition could then be complemented by more frequent data collection following initiation of antipsychotics. Of note, this group have also been shown to have less healthy diets than the general population (Labad et al. 2015; Manzanares et al. 2014) and are likely not representative of all those who will later experience psychosis.

A previous review has shown the promise of dietary interventions in psychosis in improving symptomatology, functioning, and quality of life (Aucoin et al. 2020). The authors noted a lack of rigour in reporting, particularly regarding participants' diets and their compliance with the dietary intervention. Future research could address this through use of gold‐standard measurements of dietary quality, such as the ASA‐24 (Subar et al. 2020). Measuring baseline diet in addition to this group's suggestion of high‐quality interventional designed studies and measured compliance would provide a rigorous examination of whether dietary pattern can be meaningfully changed in FEP.

### 4.5 Limitations

Our study has a number of limitations. Firstly, our study is limited by all data being self‐reported, which is a common limitation of nutritional research (Subar et al. 2015). Data may have been subject to social desirability bias, particularly as it was collected in a healthcare setting, which would have led to underreporting of unhealthy food consumption and overreporting of healthy food consumption. Secondly, our interpretation of participants' compliance with dietary guidelines is limited, as we did not ask this directly, instead inferring it from responses to related questions. We attempted to map responses to our questionnaire to the Australian guidelines, but this may have overestimated the proportion of people who met recommendations for consumption of wholemeal foods. This also meant we used two proxies for adherence to guidelines on discretionary foods, one of which was likely an overestimate and one an underestimate for compliance with this guideline. We used only Australian guidelines, which are broadly similar to guidelines in other countries, but advise on specific servings rather than general advice (Herforth et al. 2019). Thirdly, while the study contained proxies for economic status, we did not measure food security, which is a vital driver of nutritional health (Gundersen and Ziliak 2015). Fourthly, our dietary questionnaire measured only frequency of consumption of the different food types, rather than quantity. Lastly, our study took place in an affluent city, in a specialist team with high multidisciplinary team support. This included provision of dietitian support, which was not universally accessed by participants. In fact, both the intervention and control groups in the main RCT showed lower weight gain than is typical in such cohorts (O'Mahony et al. 2026). As such, our results may lack external validity.

## 5 Conclusion

Our study suggests that dietary patterns of people with first‐episode psychosis are generally unhealthy and do not change over the course of their first 6 months of treatment. The results highlight the inequity of dietary health in both people with psychosis and those in this cohort with lower levels of education. As such, dietary advice and support represent interventions with high marginal gain in this cohort and should be part of routine clinical care.

## Funding

Brian O'Mahony is an ICAT fellow and this research was funded by the Health Research Board (ICAT‐2022‐001) and the ICAT Programme, which is supported by the Health Service Executive, National Doctors Training and Planning, the Health and Social Care, Research and Development Division, the Northern Ireland Medical and Dental Training Agency, the Department of Agriculture, Food and the Marine, and the College of Anaesthesiologists of Ireland.

## Conflicts of Interest

The authors declare no conflicts of interest.

## Data Availability

The data that support the findings of this study are available on request from the corresponding author. The data are not publicly available due to privacy or ethical restrictions.

## Appendix A Dietary Questionnaire

1. **Wholegrain bread** How often do you usually eat whole grain breads or crackers?
  ○ [ ] Never or once per week
  ○ [ ] Once or twice per week
  ○ [ ] More than twice per week
2. **Wholegrain cereals** *How often do you usually eat whole grain cereals? (*e.g., *Weetbix, Porridge, muesli)*
  ○ [ ] Never or once per week
  ○ [ ] Once or twice per week
  ○ [ ] More than twice per week
3. **Seafood (not fried)** *How often do you eat fish or seafood that IS NOT fried (including tinned)?*
  ○ [ ] Never
  ○ [ ] Less than once per week
  ○ [ ] Once or twice per week
  ○ [ ] More than twice per week
4. **Low fat dairy**: *How many servings of low fat milk, cheese, or yogurt do you usually have each DAY?*
  ○ [ ] None
  ○ [ ] One
  ○ [ ] Two or more
5. **Vegetables** *How many different vegetable servings do you usually have at your main meal of the day?*
  ○ [ ] None
  ○ [ ] Less than once per week
  ○ [ ] One or two
  ○ [ ] Three or more
6. **Root vegetables**: *How often do you eat carrots or sweet potatoes?*
  ○ [ ] Never
  ○ [ ] Less than once per week
  ○ [ ] Once or twice per week
  ○ [ ] More than twice per week
7. **Salad** *How often do you eat rocket, spinach*, *or silverbeet?*
  ○ [ ] Never
  ○ [ ] Less than once per week
  ○ [ ] Once or twice per week
  ○ [ ] More than twice per week
8. **Broccoli & cauliflower** *How often do you eat broccoli or cauliflower?*
  ○ [ ] Never
  ○ [ ] Less than once per week
  ○ [ ] Once or twice per week
  ○ [ ] More than twice per week
9. **Fruit** *How often do you eat fruit (not including juice)? Please include fresh, canned or frozen fruit*
  ○ [ ] Never
  ○ [ ] Once or twice per week
  ○ [ ] Three to five times per week
  ○ [ ] Almost every day
10. **Olive oil**: *How often do you consume olive oil?*
  ○ [ ] Never
  ○ [ ] Less than once per week
  ○ [ ] Once or twice per week
  ○ [ ] More than twice per week
11. **Legumes** *How often do you consume legumes, such as lentils or chickpeas?*
  ○ [ ] Never
  ○ [ ] Less than once per week
  ○ [ ] Once or twice per week
  ○ [ ] More than twice per week
12. **Nuts** *How often do you consume fresh nuts, such as almonds, cashews, walnuts or brazil nuts?*
  ○ [ ] Never
  ○ [ ] Less than once per week
  ○ [ ] Once or twice per week
  ○ [ ] More than twice per week
13. **Soft drinks** *14. How often do you drink (non‐diet) soft drinks or cordials?*
  ○ [ ] Never
  ○ [ ] Less than once per week
  ○ [ ] Once or twice per week
  ○ [ ] More than twice per week
14. **Chocolate** *How often do you usually eat sweets or chocolate?*
  ○ [ ] Never
  ○ [ ] Less than once per week
  ○ [ ] Once or twice per week
  ○ [ ] More than twice per week
15. **Crisps** *How often do you eat chips, twisties or something similar?*
  ○ [ ] Never
  ○ [ ] Less than once per week
  ○ [ ] Once or twice per week
  ○ [ ] More than twice per week
16. **Chips** *How often do you eat pies, sausage rolls or hot chips?*
  ○ [ ] Never
  ○ [ ] Less than once per week
  ○ [ ] Once or twice per week
  ○ [ ] More than twice per week
17. **Cakes** *How often do you eat cakes, biscuits, ice creams or doughnuts?*
  ○ [ ] Never
  ○ [ ] Less than once per week
  ○ [ ] Once or twice per week
  ○ [ ] More than twice per week
18. **Deli meats**: *How often do you eat lunchmeats or deli meats (such as ham or strass), bacon or sausage?*
  ○ [ ] Never
  ○ [ ] Once or twice per week
  ○ [ ] More than twice per week
19. **Takeaway** *How often do you eat takeaway meals, such as McDonalds, Kentucky Fried Chicken, Pizza Hut or Hungry Jacks?*
  ○ [ ] Never
  ○ [ ] Once or twice per week
  ○ [ ] More than twice per week

## Appendix C Imputed Results and Plots

**TABLE C1:** Comparison of dietary patterns from baseline to 3 and 6 month follow‐up (pooled imputed data).

Comparison	3 months			6 months		
	3 m V	3 m *p*	3 m *p*(adj)	6 m V	6 m *p*	6 m *p*(adj)
Broccoli cauliflower	178.0	0.036	0.364	285.5	0.767	0.832
Cakes	526.5	0.162	0.649	604.0	0.074	0.421
Chips	442.8	0.148	0.649	534.5	0.480	0.730
Chocolate & sweets	624.2	0.078	0.518	609.8	0.005	0.102
Crisps	475.5	0.370	0.719	568.8	0.664	0.829
Deli	253.0	0.848	0.848	374.5	0.127	0.509
Fruit	353.2	0.255	0.684	227.5	0.074	0.421
Legumes	248.5	0.371	0.719	292.0	0.389	0.648
Low fat dairy	312.2	0.693	0.848	228.8	0.240	0.570
Nuts	412.0	0.590	0.848	457.5	0.540	0.730
Oliveoil	450.0	0.258	0.684	517.2	0.704	0.829
Root veg	407.5	0.395	0.719	346.2	0.901	0.901
Salad	252.8	0.274	0.684	423.2	0.548	0.730
Seafood not fried	373.5	0.749	0.848	398.2	0.790	0.832
Softdrinks	407.2	0.833	0.848	608.8	0.084	0.421
Takeaway	128.8	0.818	0.848	525.8	0.257	0.570
Vegetables	244.5	0.660	0.848	355.8	0.329	0.648
Wholegrain bread & crackers	202.8	0.557	0.848	518.5	0.388	0.648
Wholegrain cereals	309.8	0.743	0.848	367.0	0.222	0.570

*Note:* Results are pooled across 10 multiply‐imputed datasets using median *p* values. All *p* values adjusted for multiple comparisons using the Benjamini‐Hochberg (FDR) method within each timepoint. V: Wilcoxon signed‐rank V statistic (median across 10 imputations). Highlighted cells indicate *p* < 0.05 (unadjusted).
